# Integrating large language models into clinical pharmacy education: applications in perioperative medication management for gastric cancer

**DOI:** 10.3389/fmed.2025.1710500

**Published:** 2025-12-18

**Authors:** Yan Wang, Shuwei Luan, Nan Shang, Xin Zhang, Qingqing Li

**Affiliations:** 1 Department of Pharmacy, First Hospital of Shanxi Medical University, The First Clinical Medical College of Shanxi Medical University, Taiyuan, Shanxi, China; 2 Department of Pharmacy, Shanxi Bethune Hospital, Shanxi Academy of Medical Sciences, Tongji Shanxi Hospital, The Third Clinical Medical College of Shanxi Medical University, Taiyuan, Shanxi, China

**Keywords:** large language models, gastric cancer, perioperative, medication therapy management, clinical pharmacy education

## Abstract

**Objective:**

This study aims to evaluate the performance of ChatGPT-4o and DeepSeek-R1 in perioperative medication therapy management for gastric cancer, assessing their reliability and practicality as auxiliary tools in clinical pharmacy education.

**Methods:**

This study utilized a retrospective design to collate issues pertaining to perioperative medication management in gastric cancer, from which a standardized question set was developed. The set was concurrently submitted to both ChatGPT-4o and DeepSeek-R1 to generate model responses. Two independent assessors, blinded to the model sources, evaluated the outputs according to a predefined framework covering three core domains: (1) Clinical applicability, assessed via a 7-point Likert scale; (2) Information quality, evaluated using the DISCERN instrument for evidence reliability and content completeness; and (3) Readability, measured through the Flesch Reading Ease Score (FRES) and the SMOG Index.

**Results:**

In the 24-item evaluation of perioperative drug therapy for gastric cancer, both models exhibited high inter-rater reliability, with Cronbach’s *α* values of 0.880 for DeepSeek-R1 and 0.852 for ChatGPT-4o. DeepSeek-R1 demonstrated superior performance in clinical applicability (Likert score: 5.63 ± 0.94 vs. 5.10 ± 0.78, *p* < 0.001) and information quality (DISCERN score: 54.50 ± 6.71 vs. 50.56 ± 6.08), although neither model reached the excellence threshold (≥65 points). Readability assessment revealed moderately complex text difficulty, with Flesch Reading Ease scores below 30 and SMOG indices indicating a reading level of ≥17 years, which remains appropriate for undergraduate clinical pharmacy education.

**Conclusion:**

Both ChatGPT-4o and DeepSeek-R1 have demonstrated potential in addressing issues related to perioperative medication management for gastric cancer, with their generated responses showing good practical Applicability and readability suitable for the clinical pharmacy professional community. However, it should be noted that the quality of information provided by both models does not currently meet professional standards for drug therapy management. Therefore, they can be utilized as auxiliary tools for training the analytical skills of undergraduate students in clinical pharmacy, but their use should be guided by mentors.

## 1 Introduction

Human health issues necessitate the most accurate information. Since their inception, large language models (LLMs) have garnered the attention of medical institutions, with their clinical applications permeating various disciplines and specialties, including anesthesiology, oncology, imaging, nursing, and public health (1). These models also demonstrate significant advantages and competitiveness in pharmacological research and pharmaceutical development. Harnessing the robust information processing capabilities of artificial intelligence to enhance clinical pharmacy services and drive educational innovation represents a promising direction with broad prospects. Such an approach provides pharmacists with a controllable and scalable tool to support complex decision-making, thereby facilitating a smooth transition from theoretical knowledge to clinical practice.

Currently, while clinical pharmacists and pharmacy students can address queries with their specialized knowledge, their responses often depend heavily on literature reviews, textbooks, or specific drug databases for comprehensive answers. In contrast, large language models exhibit remarkable potential in this domain due to their capacity to process and analyze vast amounts of information quickly and accurately (2). However, the powerful language generation capabilities of these models may pose an inherent risk regarding the verification of information quality, a challenge consistently noted in previous studies that have emphasized their multifaceted limitations (3, 4). This tension between technological potential and real-world applicability is especially evident in clinical pharmacy, where systematic, multidimensional evaluation frameworks remain underdeveloped (5). As artificial intelligence becomes increasingly embedded in healthcare, regulatory authorities worldwide have emphasized transparency, safety, and accountability as essential preconditions for clinical implementation (6). Accordingly, developing evaluation frameworks that align with these governance principles is critical for ensuring responsible AI deployment.

In this context, selecting diseases that encompass a wide range of clinical pharmacy practice scenarios and are highly dependent on individualized decision support as research subjects allows for a more comprehensive evaluation of the performance of large language models in real-world clinical settings. Perioperative pharmacotherapy management for gastric cancer can serve as a representative research entry point. This stage involves multiple medications and numerous complex decisions related to treatment continuity, resulting in a high demand for pharmacist guidance. Moreover, gastric cancer imposes a significant disease burden in China, with newly diagnosed cases and related deaths accounting for approximately 37.0 and 39.4% of the global totals, respectively (7, 8). Implementing precise pharmacotherapy, particularly in the context of complex medication regimens to prevent, identify, and intervene in drug-related issues, has become an important challenge and opportunity for improving survival rates and quality of life.

Consequently, this study concentrates on the perioperative context of gastric cancer, thoroughly assessing the response performance of two large language models in medication management within this scenario from three dimensions. The findings aim to promote the scientific and responsible integration of large language models into clinical pharmacotherapy, while providing empirical evidence to support the refinement of relevant AI governance and healthcare policy frameworks. At the same time, this research constitutes an important and prudent step toward advancing the application of large language models in higher clinical pharmacy education.

## 2 Methods

### 2.1 Model selection and rationale

Model selection was guided by prior peer-reviewed evaluations that demonstrated distinct performance characteristics and architectural differences among leading LLMs. ChatGPT-4o was selected as a general-purpose benchmark model, given its widespread adoption in medical education and clinical reasoning research (9). In contrast, DeepSeek-R1 was included as a domain-adaptive model with demonstrated superiority in gastroenterology-specific evaluations (10).

To facilitate a transparent and comprehensive comparison, the technical specifications of ChatGPT-4o and DeepSeek-R1 are summarized in Table 1 (11–14). Briefly, ChatGPT-4o (OpenAI, USA) is a multimodal, general-purpose LLM trained through reinforcement learning from human feedback (RLHF) and multimodal fine-tuning to enhance contextual understanding and response coherence. In contrast, DeepSeek-R1 (DeepSeek AI, China) is a reasoning-optimized, search-augmented model built on a Mixture-of-Experts (MoE) architecture and aligned via large-scale reinforcement learning. This selection enables comparison between general and search-augmented LLM frameworks within the context of perioperative pharmacotherapy.

**Table 1:** Technical specifications of the evaluated large language models.

Feature	GPT-4o	DeepSeek-R1
Underlying Architecture	Dense Transformer (exact architecture not disclosed)	Mixture of Experts (MoE)
Context Window	128,000 tokens	128,000 tokens
Model Type	Proprietary, Multimodal	Open-source (weights available), Text-only
Parameter Count	Not publicly disclosed	236 Billion Total (21 Billion Active)
Key Training/Alignment	Supervised Fine-Tuning (SFT), Reinforcement Learning from Human Feedback (RLHF)	Supervised Fine-Tuning (SFT), Grouped Query Attention (GQA)
Knowledge Cut-off	October 2023	July 2024

The two generative biglanguage models used in this study, ChatGPT-4o and DeepSeek-R1, are a publicly available resource and therefore exempt from institutional ethics review board review.

### 2.2 Question design and validation

The 24-question set was developed through a structured process combining real-world clinical pharmacy cases and standardized educational simulations.

General Questions (*n* = 20): These questions were derived from pharmaceutical consultation and intervention cases collected during ward rounds from March 17 to April 17, 2025. Using the Pharmaceutical Care Network Europe (PCNE) classification system, we analyzed influencing factors and identified high-frequency problem types and their contributing factors, which were consolidated into representative perioperative medication management scenarios. The PCNE analysis results are presented in Supplementary Tables 1, 2.

Individualized Questions (*n* = 4): Developed from standardized patient (SP) cases used in the clinical pharmacy internship assessment question bank, these questions simulated complex, individualized pharmacotherapy decisions.

The questions were developed by an expert panel (two clinical pharmacists, one gastrointestinal surgeon, and one gastrointestinal surgery nurse) and externally reviewed by two senior pharmacy educators. Answer references were based on official drug inserts and the latest guideline recommendations. The complete question set and supporting reference materials are provided in Supplementary Table 3.

### 2.3 Model evaluation procedures

The 24 standardized prompts were entered into the web interfaces of ChatGPT-4o and DeepSeek-R1 on April 28, 2025, to minimize potential performance variation due to system updates. To ensure consistency, all questions were entered into ChatGPT-4o and DeepSeek-R1 separately by one student, with each prompt initiated in a new independent session to avoid contextual interference, and the first generated responses were recorded. All responses were evaluated by two independent reviewers, one associate gastrointestinal surgeon and one surgical pharmacist, who scored the model-generated answers individually based on their academic backgrounds and who were unaware of each other’s answers to prevent potential bias. Each reviewer independently evaluated the responses generated by the model using a predefined framework for assessing medical LLMs. The framework assesses model performance across three critical dimensions: applicability, information quality, and readability. To ensure an objective and multidimensional evaluation, four well-established instruments were employed: a 7-point Likert scale to assess applicability, the DISCERN tool for evaluating information quality, and two validated readability metrics—the Flesch Reading Ease score and the SMOG (Simple Measure of Gobbledygook) index.

#### 2.3.1 Applicability analysis

The Applicability of the answers generated by the two models was evaluated using a 7-point Likert scale (15), where 1 represents “not at all practical” and 7 signifies “completely practical.” This scale is presented in Table 2 of our study. Following the guidelines and consensus on perioperative drug therapy and pharmacological monitoring for gastric cancer, we screened and identified key information from drug inserts and other relevant standards. Each model’s answers were meticulously compared to this key ground truth, and subjective evaluations were collected across multiple dimensions, including completeness, accuracy, and extensiveness of the information. This process provided a quantitative basis for optimizing the models. To ensure the internal consistency of the ratings provided by the audit experts, a confidence analysis was conducted.

**Table 2:** Applicability score - Likert scale.

Score	Scoring criteria
1	Not applicable at all: Response not relevant to the question, and missing important information.
2	Not applicable: Very limited use, most important information is missing or incorrect.
3	Relatively Not applicable: Most important information is mentioned, but some important information is incomplete or incorrect.
4	Generally applicable: Covers the most important information, still some important information is missing or incorrect.
5	Relatively applicable: Most important information is addressed, but some important information is still incomplete or incorrect.
6	Applicable: All important information is addressed, information and details are correct.
7	Completely applicable: All important information was addressed correctly, and additional information and resources were provided.

#### 2.3.2 Quality analysis

The quality of each answer was assessed using the validated DISCERN scoring system, developed by D. Charnock, which is widely recognized for evaluating the quality of health information. This tool is particularly useful for assessing written health information related to treatment choices (16, 17). The DISCERN system measures the quality of health information by evaluating the source’s ability to provide accurate and current information regarding treatment options, outcomes, and uncertainties. It consists of three tiers: (1) reliability of information (8 questions), (2) completeness and accuracy concerning drug treatment information (7 questions), and (3) a composite quality rating. Each question’s quality was scored on a scale from 1 to 5, culminating in a total possible score of 80. Higher scores indicate a greater overall quality of information, with categorizations as follows: excellent (excellent = 63–75, good = 51–62, fair = 39–50, poor = 27–38, and very poor = 15–26). Two independent reviewers evaluated each response and calculated the average score. The DISCERN scoring system was slightly modified for our study, with the specific modifications detailed in Table 3.

**Table 3:** DISCERN scoring system.

DISCERN questionnaire
Section 1. Are the responses reliable?
1. Are the objectives clear?
2. Were the objectives achieved?
3. Was the objective met?
4. Is it clear what sources of information (other than the questioner) were used to develop the response?
5. Is it clear when the information used or reported in the response was generated?
6. Is it fair and unbiased?
7. Does it provide details of support and sources of information?
8. Does it refer to areas of uncertainty?
Section 2. What is the quality of the information on treatment options?
9. Does it describe each treatment?
10. Does it describe the benefits of each treatment?
11. Does it describe the risks of each treatment?
12. Does it describe what will happen if the treatment is not administered?
13. Does it describe how treatment choices affect overall quality of life?
14. Is it clear that there may be more than one possible treatment option?
15. Does it provide support for shared decision making?
Section 3. Overall rating of responses
16. Rate the overall quality of responses as a source of information based on answers to all of the above questions.

#### 2.3.3 Readability analysis

To evaluate the readability of the text generated by the Big Language Model, we employed two quality assessment tools: the Flesch Reading Ease Score (FRES) (18) and the Simple Measure of Gobbledygook (SMOG) Index (19). These tools assess both readability and text complexity. FRES has a scoring range from 0 to 100 points, where scores between 0 and 30 indicate difficult readability, scores from 30 to 50 are hard, scores from 50 to 60 are moderately difficult, scores between 70 and 80 are easy, and scores from 80 to 100 are very easy. Higher scores denote easier readability, while lower scores indicate more challenging text. The FRES is calculated based on the average sentence length and the average number of syllables per word, as detailed in the following formula:


$$206.835-1.015\;(\frac{\text{total words}}{\text{total sentences}})-84.6\;(\frac{\text{total syllables}}{\text{total words}})$$

The SMOG Index is a tool used to estimate the minimum educational level required for a reader to comprehend a given text. It achieves this by analyzing the number of multi-syllabic words and the average length of sentences. A higher SMOG score indicates that the text is more challenging to read and understand, whereas a lower score suggests that the content is more accessible. The formula for calculating the SMOG Index is as follows:


$$\text{grade}=1.0430\;\sqrt{\text{number of polysyllables}\times \frac{30}{\begin{array}{c} \text{number of}\;\\ \text{sentences} \end{array}}}+3.1291$$

### 2.4 Statistical analysis

The data from this study were statistically analyzed using SPSS version 25.0. In the descriptive analysis, data were expressed as mean ± standard deviation. Reliability analysis assessed the internal consistency of the reviewing experts through the Cronbach’s alpha coefficient and the 95% confidence interval (CI). The Cronbach’s alpha ranges from 0 to 1, with values closer to 1 indicating a higher level of internal consistency and reliability. A Cronbach’s *α* coefficient above 0.7 is generally accepted as indicative of good reliability, while a coefficient below 0.6 suggests insufficient internal consistency reliability. The Shapiro–Wilk test was employed to evaluate whether the variables exhibited a normal distribution. Independent samples t-tests were conducted to compare the overall differences between the two models, while paired samples t-tests were utilized to assess between-group differences among the two rating experts. The significance level for this study was established at *p* < 0.05.

## 3 Results

### 3.1 Applicability analysis

#### 3.1.1 Reliability analysis

This study evaluated the consistency of the scoring records provided by the two reviewers regarding the content of the answers generated by the large language model. The evaluation was conducted using the Cronbach’s *α* coefficient along with the calculations of the 95% confidence interval (CI). The Cronbach’s α value for DeepSeek-R1 was found to be 0.880 (95% CI, 0.313–1.000), while ChatGPT-4o’s Cronbach’s α value was 0.852 (95% CI, 0.148–1.000). These results indicate a high level of agreement between the two reviewers.

#### 3.1.2 Likert scale results

The results of the paired t-test indicate that the Likert Applicability scores for ChatGPT-4o, as assessed by the two reviewers, were 5.25 ± 0.74 and 4.96 ± 0.81, respectively. In contrast, the mean scores for DeepSeek-R1 were 5.79 ± 1.02 and 5.46 ± 0.83, respectively. Statistically significant differences in the Applicability scores were observed (*p* < 0.05). The overall means were further analyzed using an independent samples t-test, which also revealed significant differences, with means of 5.1 ± 0.78 and 5.63 ± 0.94 for the two models, reflecting a Likert scale grading of relative Applicability. The detailed results are presented in Table 4.

**Table 4:** Results of Likert scale data analysis.

Applicability	Chatgpt 4o	DeepSeek-R1	*p*	T (95% Cl)
Rater 1	5.25 ± 0.74	5.79 ± 1.02	0.002	3.406 (0.159–0.213)
Rater 2	4.96 ± 0.81	5.46 ± 0.83	0.003	3.391 (0.195–0.805)
Mean	5.1 ± 0.78	5.63 ± 0.94	*p* < 0.001	4.854 (0.305–0.737)

Likert scale scores range from 1 to 7 (1 being not at all useful and 7 being completely useful).

### 3.2 Results of quality analysis

**Figure 1:**
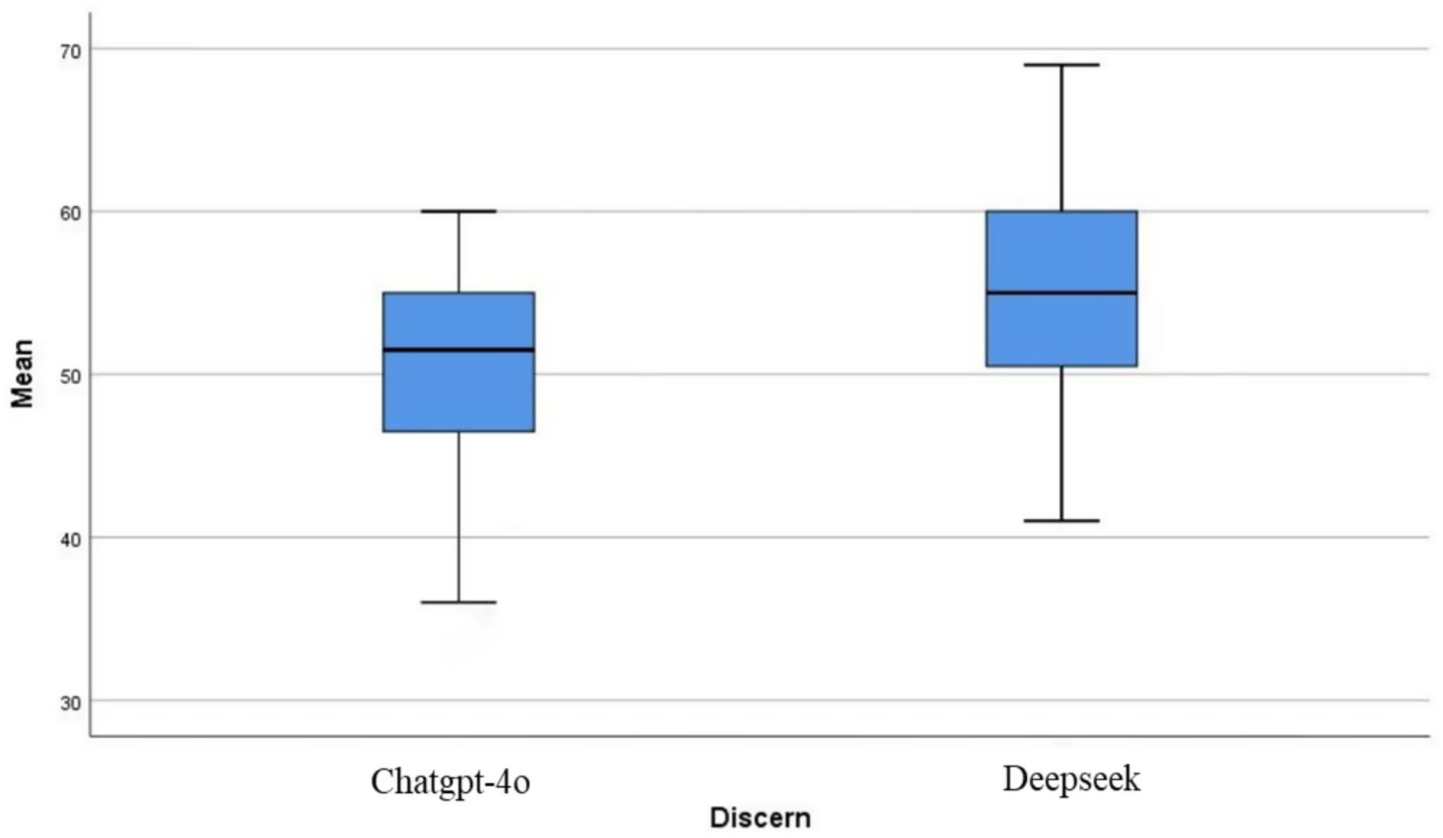
DISCERN scoring system means.

In the quality assessment phase of this study, we employed the DISCERN tool to systematically evaluate the information quality of the generated answers to 24 perioperative drug therapy questions pertaining to gastric cancer. The analysis revealed that the mean score for ChatGPT-4o was 50.56 ± 6.08, indicating that the quality of the information provided was at an average level. In contrast, the mean score for DeepSeek-R1 was 54.50 ± 6.71, which meets the criteria for good information quality (Figure 1). An independent samples t-test demonstrated that the difference in scores between the two groups was statistically significant (*p* < 0.05), thereby confirming the existence of differences in information quality between the two models within the DISCERN assessment framework.

Separate analyses of the Discern scale score entries for the two models were conducted, revealing that six entries had mean scores of less than 3 for both models: T4, T5, T7, T8, T10, and T13 (see Table 5). T4 assesses whether it is clear which sources of information have been utilized in composing the response, in addition to the questioner. T5 similarly evaluates the clarity of the sources of information used for the response. T7 examines whether the response provides details regarding support and information sources. All of these items pertain to the sourcing of information in the answers, and it is noteworthy that much of the output text lacks annotations regarding the sources, the timing of their production, or specific evidence. T8 assesses whether the response addresses areas of uncertainty, T10 evaluates whether it describes the benefits of each treatment, and T13 considers how treatment choices can impact overall quality of life. These findings indicate a limitation in the comprehensiveness of the information quality provided.

**Table 5:** DeepSeek-R1 and ChatGPT-4o response quality analysis.

LLM	T1	T2	T3	T4	T5	T6	T7	T8	T9	T10	T11	T12	T13	T14	T15	T16
DeepSeek-R1	4.96	4.13	4.90	1.85	1.21	5.00	1.56	2.27	4.50	2.63	4.13	3.21	0.48	4.88	4.75	4.02
ChatGPT-4o	4.85	3.67	4.71	1.08	0.08	5.00	0.79	2.29	4.42	2.65	4.02	3.19	0.50	4.85	4.56	3.67

### 3.3 Readability results

**Figure 2:**
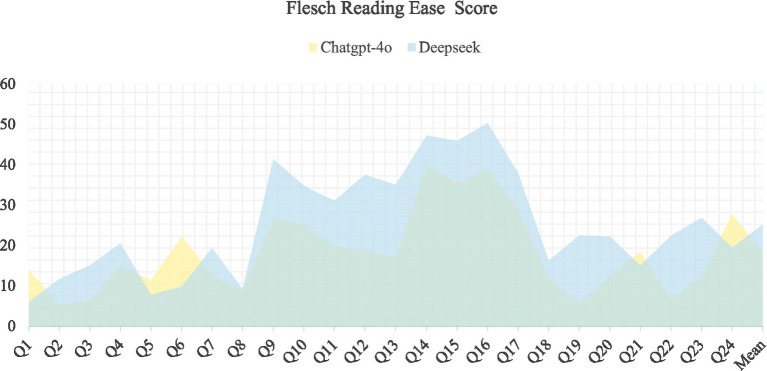
Flesch reading ease analysis.

**Figure 3:**
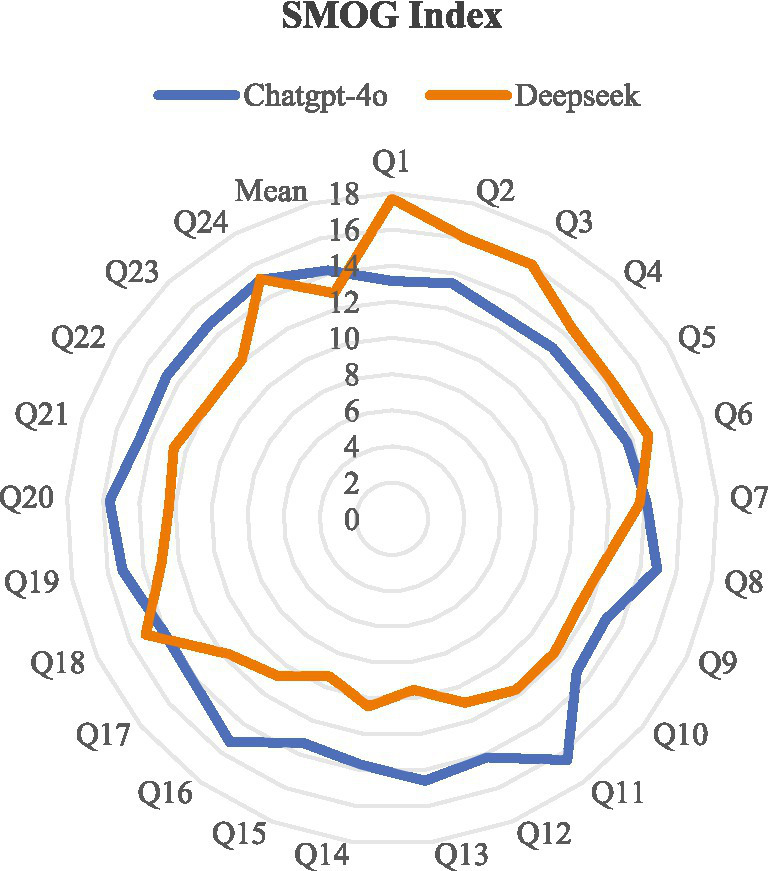
SMOG analysis.

In the final phase of this study, we employed two well-established readability assessment tools: the Flesch Reading Ease Score (FRES) and the Simple Measure of Gobbledygook (SMOG) Index, to analyze the answer texts generated by the ChatGPT-4o and DeepSeek-R1 models. The FRES scoring results (Figure 2) indicate that the average score for ChatGPT-4o is 18.48 ± 10.28, while the average score for DeepSeek-R1 is 25.28 ± 13.29. According to the FRES rubric, both models scored well below 30, which clearly categorizes their generated texts as falling within the ‘Very Difficult’ level. Furthermore, the SMOG index analysis (Figure 3) provides an additional dimension of corroboration: the mean score for ChatGPT-4o was 14.17 ± 0.96, compared to 12.88 ± 2.13 for DeepSeek-R1. The SMOG scores intuitively correlate text readability to the required U. S. educational grade level. These scores suggest that comprehending the content of the model-generated answers theoretically necessitates readers to possess reading abilities ranging from the 11th grade and above in high school to college level, i.e., suitable for a demographic of readers aged 17 and older. This finding reconfirms the text’s high reading threshold; however, the level of difficulty is deemed appropriate and acceptable for the target group, particularly undergraduate clinical pharmacy students.

In summary, DeepSeek-R1 demonstrated a slight advantage over ChatGPT-4o across four scores within the three dimensions (see Table 6). However, the quality of responses from both models was inadequate, failing to meet the essential requirements for pharmacy education in the specific context of perioperative medication management for gastric cancer.

**Table 6:** Results of the analysis of the three dimensions.

Evaluation tool	Chat gpt 4o (Mean±SD)	DeepSeek-R1 (Mean±SD)	*p*	*t* (95% Cl)
Likert	5.10 ± 0.78	5.63 ± 0.94	*p* < 0.001	4.854 (0.305, 0.737)
DISCERN	50.56 ± 6.08	54.50 ± 6.71	*p* < 0.001	4.189 (2.047, 5.828)
FRE	18.48 ± 10.28	25.28 ± 13.29	*p* < 0.001	3.858 (3.15, 10.434)
SMOG	14.17 ± 0.96	12.88 ± 2.13	*p* = 0.021	2.481 (0.218, 2.376)

### 3.4 Analysis of representative critical failures

Among the 24 evaluated queries regarding perioperative pharmacotherapy in gastric cancer, LLM-generated responses exhibited several limitations. Identified issues included omissions, factual inaccuracies, conceptual or mechanistic misunderstandings, overgeneralization in clinical recommendations, failure to account for individualized variables, lack of traceability, imbalanced risk–benefit reporting, and uncertainty recognition failure. Lack of traceability was among the most frequent, while omissions and uncertainty recognition were also commonly observed (see Table 7). Overall, these findings indicate that although LLMs can provide comprehensive guidance, verification by mentors remains essential to ensure accuracy and suitability for individual patients in clinical practice.

**Table 7:** Typical errors and limitations of LLM responses in perioperative pharmacotherapy in gastric cancer.

Category	Percentage	Description of error	Typical examples
Omission	3/24	LLM responses list incompatibilities by drug category, leading to incomplete coverage when many agents are involved.	For cefuroxime sodium, the model-generated output failed to mention two clinically uncommon but documented incompatible agents, namely polymyxin B sulfate and methylphenidate
	3/24	When listing examples within a drug class, LLMs often omit newly marketed agents	In the context of perioperative management of SGLT-2 inhibitors, the model listed dapagliflozin, canagliflozin, and empagliflozin as requiring discontinuation 3 days before surgery, but failed to mention that ertugliflozin requires a 4-day preoperative discontinuation.
Factual inaccuracy	1/24	Incorrect statements regarding therapeutic guidance	In perioperative prophylactic antibiotic recommendations for patients with beta-lactam allergy, ChatGPT-4o suggested aztreonam or quinolones, while DeepSeek recommended clindamycin plus gentamicin. Both recommendations are inconsistent with clinical guidelines, which suggest vancomycin, teicoplanin, or clindamycin for Gram-positive coverage, and aztreonam, fosfomycin, or aminoglycosides for Gram-negative coverage.
	1/24	Incorrect statements about preoperative discontinuation	For sulfonylureas, the response suggested adjusting the dose preoperatively instead of correctly recommending discontinuation 24 h before surgery.
Conceptual or mechanistic misunderstandings	1/24	Misinterpretation of pharmacokinetic principles	When asked about the incompatibilities of injectable ceftazidime, the response stated that PPIs (e.g., omeprazole) or antacids (e.g., sodium bicarbonate), which alter gastric pH, might affect the absorption of intravenously administered ceftazidime.
	1/24	Confusion between dosage forms	The response included insulin in the list of postoperative oral antidiabetic agents to be restarted, failing to distinguish between injectable and oral formulations
Overgeneralization in clinical recommendations	5/24	overly broad recommendations.	Recommendations for inhalation therapy monitoring were comprehensive but lacked specific drug dosing information, failing to provide actionable guidance.
Failure to account for individualized variables	4/24	Advice lacks tailoring to specific patient conditions.	Models stated that NOACs should be stopped 72 h preoperatively for gastric cancer surgery but failed to adjust guidance for impaired renal function, which may require longer cessation.
Lack of traceability	20/24	Missing traceable references, inaccuracy, and evidence level	responses lacked source annotation, update timelines, or evidence hierarchy
Imbalanced risk–benefit information	4/24	Benefits underreported relative to risks	While adverse effects/contraindications were discussed, key benefits (e.g., survival improvement, symptom relief, QoL impact) were insufficiently reported.
Uncertainty recognition failure	7/24	Failure to mark areas lacking clinical consensus	Models did not flag treatment options where clinical consensus is evolving, failing to acknowledge uncertainty domains.

## 4 Discussion

The primary advantages of generative macrolanguage modeling, which has seen rapid development in the medical field, include its extensive domain knowledge, proficiency in understanding specialized medical terminology and concepts, and robust contextual comprehension and reasoning abilities. These features facilitate effective human-computer interaction, enable responses to open-ended questions, and support the generation of coherent and fluent natural language (20). However, for online information to be clinically useful, it must be practical, reliable, applicable, and easily understandable, which is why our study implemented a quantitative multidimensional evaluation framework to comprehensively assess these key dimensions.

This study advances the evaluation of large language models (LLMs) in perioperative medication management through methodological innovations. First, the assessment framework is grounded in real-world pharmacist consultation and intervention cases, ensuring that challenges reflect authentic clinical practice. By directly linking performance metrics to real-world decision-making scenarios, it effectively addresses the limitations of previous studies that primarily relied on question banks and pharmaceutical databases (21, 22). Second, the research employs the European Patient Care Network (PCNE) classification system to systematically analyze influencing factors and identify high-frequency perioperative medication issues. Building on these data, clinically representative test scenarios are further supplemented to establish a reproducible, evidence-based framework for evaluating complex medication treatment scenarios. This approach transcends the limitations of traditional accuracy-focused benchmarking, enabling systematic assessment of challenges in long-term clinical monitoring—issues rarely addressed in earlier studies (23, 24).

The test results of two readability analysis tools show that the difficulty of the text is mainly due to the use of pharmaceutical terminology, rather than the complex sentence structure. This level of difficulty, characterized by specialized content, falled within the acceptable range of the knowledge base and cognitive abilities of undergraduate clinical pharmacy students, indicating that the design is appropriately matched to the target audience.

This study further evaluated the capabilities of two widely applicable large language models in managing perioperative drug therapy for gastric cancer through comparative analysis. The results indicated that both ChatGPT-4o and DeepSeek-R1 were relatively effective in addressing pertinent information; however, some critical details were either incomplete or incorrect. The completeness of responses from ChatGPT-4o and DeepSeek-R1 was limited when faced with numerous medicinal combinations, as their responses were organized by drug classification. For example, among the drugs that are incompatible with cefuroxime, two agents that are not commonly used in clinical practice—polymyxin B sulfate and methylphenidate—were not mentioned in the responses generated by the language models. In addition to such omissions, factual inaccuracies were also observed. When asked about the incompatibilities of injectable ceftazidime, ChatGPT-4o incorrectly stated that concomitant use with proton pump inhibitors (e.g., omeprazole) or antacids (e.g., sodium bicarbonate) could reduce ceftazidime absorption by increasing gastric pH. This explanation reflects a misunderstanding of pharmacokinetic principles, as ceftazidime is administered intravenously and thus bypasses gastrointestinal absorption entirely. These examples illustrate both gaps in knowledge coverage and conceptual reasoning errors that may have direct implications for clinical decision-making. Similarly, in questions regarding complications or prophylactic medication, the models’ recommendations for drug administration or discontinuation tended to be generic and failed to account for specific pathological conditions. For example, while guidelines suggest that novel oral anticoagulants should generally be discontinued 72 h prior to surgery in patients with gastric cancer, those with renal impairment require a longer discontinuation period. Moreover, the exact timing varies depending on the degree of renal dysfunction and the specific anticoagulant used. Such oversimplified guidance highlights the models’ limitations in providing individualized clinical recommendations. Nevertheless, not all model outputs were inferior to the reference standards. The research process revealed that DeepSeek-R1, in particular, provided additional pertinent details beyond those contained in the standard answers. For example, it correctly identified the contraindication of mixing ceftazidime and aminoglycosides within the same delivery system or syringe and recommended administering them at intervals exceeding 1 hour or through different infusion routes. Such insights indicate that, despite existing limitations, LLMs may occasionally capture clinically relevant nuances that are consistent with pharmacological reasoning and could potentially enhance the comprehensiveness of decision support in perioperative medication management.

To mitigate assessment bias, the quality of information was further evaluated using the DISCERN questionnaire. To further elucidate the findings derived from the DISCERN-based quality assessment, we conducted a detailed analysis of the specific deficiencies identified in model-generated responses. Firstly, the generated responses typically exhibited a lack of traceability, as evidenced by DISCERN scores related to the source of information, which ranged from a mere 0.08 to 1.85 out of 5. This was primarily due to the observation that over 80% of the responses failed to annotate the source of information, did not specify a time frame for knowledge updates, and lacked a description of the level of evidence, which could potentially affect the verifiability of the information provided. Secondly, while risk-related discourses, such as adverse effects and contraindications, were frequently mentioned, information on treatment benefits, including survival outcomes and symptomatic relief rates, was less comprehensive, and discussions of the impact on quality of life were particularly limited. This imbalance may lead to misleading risk–benefit assessments in clinical decision-making. Furthermore, the model did not consistently identify and categorize as uncertainty domains’ those treatment options where consensus in clinical practice is still evolving. This aspect requires attention, as it may not fully reflect the current limitations of medical knowledge and could potentially obscure ambiguities in key information, which might contribute to challenges in clinical decision-making. Our quality assessment results align with previous studies, evaluating five mainstream medical models: MEDITRON (25), Mistral (26), Llama 3 (27), GPT-4 (28), and Claude-3.5 (29). Even the most advanced large language models (LLMs) struggle to handle complex clinical tasks when provided with limited contextual clues. Our study further demonstrates that, although current large language models (LLMs) exhibit remarkable natural language fluency and reasoning capabilities, their domain adaptability and knowledge verification remain limited. Technically, these limitations include insufficient mechanisms for source tracing, evidence hierarchy integration, inadequate incorporation of multidimensional clinical indicators in generated content, limited integration with authoritative evidence-based databases, and the lack of ability to identify the boundary of medical knowledge. Overcoming these constraints requires not only continuous optimization of model architectures and alignment strategies but also targeted fine-tuning using authoritative evidence-based medical datasets to ensure factual accuracy and clinical safety.

Future research should focus on developing comprehensive, multi-dimensional strategies for model optimization. Hierarchical knowledge integration using structured knowledge graphs can encode fundamental biomedical facts, clinical evidence, guideline recommendations, and domains of consensus uncertainty, enabling models to reason across diverse clinical indicators and evidence levels. To ensure traceability, retrieval-augmented generation or evidence-chain reasoning techniques can be applied. By integrating external knowledge sources, trained models can be dynamically linked with continuously updated authoritative pharmaceutical databases such as Micromedex® and ClinicalPharm®, with each generated statement automatically annotated with source references, evidence grades, and timestamps (30). This mechanism addresses both the timeliness of knowledge and the requirements of evidence-based traceability. Based on domain-specific pretraining and dynamic validation, task-oriented fine-tuning guided by reinforcement learning from human/expert feedback (RLHF) can further align model outputs with clinical reasoning requirements (31). Fine-tuning techniques also effectively expand data coverage and professional boundaries in core tasks such as medication therapy management, ensuring more precise responses that meet the complex needs of clinical pharmacy services. Training should emphasize enhancing professional concept comprehension and clinical reasoning ability. Directed autoregressive training on such specialized clinical pharmacy corpora ensures stable outputs in downstream tasks, including prescription review, medication education, and adverse drug reaction prediction.

As these technical advances evolve, regulatory frameworks are simultaneously shaping the expectations for transparency and safety, which further informs the direction of future model development and evaluation. The European Union’s Artificial Intelligence Act (Regulation (EU) 2024/1689) establishes a unified regulatory framework emphasizing transparency, safety, and traceability in AI systems. It mandates that high-risk and general-purpose large language models (LLMs) disclose essential information, including model architecture, training data, evaluation outcomes, and update strategies, to ensure controllability and reliability in critical domains (6). Future evaluations will extend beyond model performance to include regulatory compliance, encompassing risk classification, adversarial testing, and traceability verification (32). Such integration will help ensure the safe, standardized, and trustworthy application of LLMs in clinical pharmacy education and practice while supporting ongoing innovation.

Future advancements in LLMs, beyond overcoming challenges such as the scarcity of high-quality domain-specific data and the alignment and integration of deep professional knowledge, will hinge critically on addressing the limited interpretability of these “black-box” models—a key step toward developing trustworthy clinical artificial intelligence. The emerging trend involves the development of domain-customized model architectures, which either encode core medical knowledge and other mathematical models natively into neural networks or adopt symbolic–neural hybrid architectures, wherein symbolic systems handle rigorous logical reasoning while neural networks manage pattern recognition and high-dimensional optimization (32, 33). Such deep integration is expected to significantly enhance task consistency and predictive accuracy, while alleviating the bottlenecks in model generalizability and trustworthiness.

## 5 Limitations

The limitations of this study include the relatively small sample size of assessors, which may introduce shared biases and reduce the representativeness of expert clinical judgment. Although the limited scope of assessment improved internal consistency among reviewers, it may not fully capture the diversity of clinical reasoning patterns. Future studies should expand the assessor pool through multidisciplinary collaboration and employ more robust consensus methodologies, such as multi-round and blinded Delphi processes, to enhance the validity and reliability of expert evaluation.

Additionally, this study was confined to the evaluation of two large language models (LLMs), which constrains the generalizability of the findings across other model architectures and versions. Future research should include a broader range of state-of-the-art LLMs to enable comparative benchmarking and to better understand cross-model variability in clinical reasoning performance.

It is also important to note that this study intentionally focused on perioperative drug therapy management in gastric cancer as a representative scenario, rather than encompassing the full spectrum of clinical contexts. While this focus may limit the generalizability of conclusions, it aligns with the exploratory design principle of anchoring on core, high-risk clinical questions. The applicability of the findings to atypical or rare drug treatment pathways remains to be validated through ongoing research. To this end, we are developing an extended question set to facilitate the translation of this technology from baseline validation toward real-world clinical implementation.

## 6 Conclusion

This study not only validated the potential of two large language models (LLMs) in clinical pharmacy applications but also revealed their core professional and technical limitations. At the current stage, to overcome these constraints, we have established a multi-dimensional evaluation framework to systematically assess the value of LLMs in clinical pharmacy education. By leveraging high-quality, task-specific data extracted from perioperative medication management for gastric cancer, this approach can significantly enhance the accuracy of personalized therapeutic recommendations and the effectiveness of medication safety alerts, while addressing the needs for scenario-specific adaptability and decision-making interpretability. This innovative methodology not only improves clinical safety but also lays the groundwork for future research based on a hierarchical optimization framework, promoting the responsible integration of LLMs into pharmacy education—supporting students in developing critical thinking and risk-assessment skills, and elevating the models from passive knowledge repositories to active decision-support systems.

## Data Availability

The original contributions presented in the study are included in the article/Supplementary material, further inquiries can be directed to the corresponding author.
