# Supplementary material for: Integrating large language models into clinical pharmacy education: applications in perioperative medication management for gastric cancer
Source: Front Med (Lausanne). 2025 Dec 18;12:1710500. doi: 10.3389/fmed.2025.1710500 (PMC12756437; doi:10.3389/fmed.2025.1710500)
Supplement: Supplementary file 1 [file Data_Sheet_1.PDF]

Supplementary Appendix

Table S1. Types of drug-related problems（DRPs）

**Primary Domain**	**Primary Domain**	**Primary Domain**	**Quantity**	**Percentage (%)**
**1.Treatment effectiveness** There is a (potential) problem with the (lack of) effect of the pharmacotherapy	**P1.1**	No effect of drug treatment despite correct use	7	10.18%
	**P1.2**	Effect of drug treatment not optimal	23	33.6%
	**P1.3**	Untreated symptoms or indication	18	26.42%
**2. Treatment safety** Patient suffers, or could suffer, from an adverse drug event. *N.B.* If there is no specific cause, skip Causes coding	**P2.1**	Adverse drug event (possibly) occurring	14	20.37%
**3. Other**	**P3.1**	Unnecessary drug-treatment	4	6.65%
	**P3.2**	Unclear problem/complaint. Further clarification	2	2.78%

Table S2. Reasons for drug-related problems（DRPs）

**Primary Domain**	**Code V9.1**	**Cause**	**Quantity**	**Percentage (%)**
**1. Drug selection** The cause of the (potential) DRP is related t of the drug (by patient or o the selection health professional)	**C1.1**	Inappropriate drug according to guidelines/formulary	12	10.43%
	**C1.2**	No indication for drug	2	1.74%
	**C1.5**	No or incomplete drug treatment in spite of existing indication	27	23.48%
**3. Dose selection** The cause of the DRP is related to the selection of the dose or dosage	**C3.1**	Drug dose too low	5	4.35%
	**C3.3**	Dosage regimen not frequent enough	53	46.09%
**4. Treatment duration** The cause of the DRP is related to the duration of treatment	**C4.1**	Duration of treatment too short	8	6.96%
	**C4.2**	Duration of treatment too long	2	1.74%
**8. Patient transfer related** The cause of the DRP can be related to the transfer of patients between primary, secondary and tertiary care, or transfer within one care institution.	**C8.1**	Medication reconciliation problem	6	5.22%

Table S3. Evaluation Question Set for Perioperative Medication Management in Gastric Cancer (24 Items)

No.	Question	Reference Source
**Section 1. Incompatibilities and Drug Interactions**		
1	As a clinical pharmacist, describe the incompatibilities of cefuroxime sodium for injection and explain the rationale for each category.	Esseti Farmaceutici S.r.l. *Cefuroxime Sodium for Injection, Drug Insert*.
2	As a clinical pharmacist, describe the incompatibilities of ceftazidime for injection, categorized by reaction type and underlying mechanism.	GlaxoSmithKline Manufacturing S.p.A. *Ceftazidime for Injection, Drug Insert*.
3	As a clinical pharmacist, describe the incompatibilities of cefotaxime sodium for injection, categorized by reaction type and cause.	Xiangbei Wellman Pharmaceutical Co., Ltd. *Cefotaxime Sodium for Injection, Drug Insert*.
4	As a clinical pharmacist, summarize the incompatibilities of ceftriaxone sodium for injection, emphasizing ionic incompatibilities and contraindicated diluents.	Shanghai Roche Pharmaceutical Co., Ltd. *Ceftriaxone Sodium for Injection, Drug Insert*.
5	As a clinical pharmacist, identify the incompatibilities of cefoperazone–sulbactam for injection and explain the rationale for each category.	Pfizer Inc. *Cefoperazone–Sulbactam for Injection, Drug Insert*.
6	As a clinical pharmacist, describe the drug interactions of vancomycin hydrochloride for injection and specify implicated agents and management strategies.	VIANEX S.A.—Plant C. *Vancomycin Hydrochloride for Injection, Drug Insert*.
7	As a clinical pharmacist, describe the drug interactions of meropenem for injection and specify implicated agents and management strategies.	Sumitomo Pharma Co., Ltd., Oita Plant. *Meropenem for Injection, Drug Insert*.
**Section 2. Therapeutic Regimens and Monitoring Essentials**		
8	Develop a perioperative prophylactic antibiotic regimen for a patient undergoing elective radical gastrectomy (Class II incision).	*Guiding Principles for Clinical Application of Antimicrobial Agents* (2015 Edition).
9	Formulate a perioperative anticoagulant management plan, including preoperative withdrawal, bridging therapy, and postoperative resumption.	*Guidelines for the Prevention and Management of Perioperative Thrombosis in General Surgery*.
10	How should perioperative management be	*Multidisciplinary Expert Consensus*

No.	Question	Reference Source
	approached in gastric cancer patients receiving long-term antithrombotic therapy?	*on Perioperative Management of Antithrombotic Therapy.*
11	How should postoperative venous thromboembolism (VTE) prevention and management be performed in gastric cancer surgery?	Same as above.
12	Develop a comprehensive perioperative pain management strategy for gastric cancer patients, including key pharmacological monitoring points.	*Expert Consensus on Postoperative Pain Management in Adults.*
13	How should analgesic dosages be adjusted for gastric cancer patients with hepatic or renal impairment during the perioperative period?	*Guidelines for Clinical Pharmacist Postoperative Pain Management.*
14	Establish a comprehensive perioperative nutritional management process for gastric cancer patients, including screening, assessment, nutritional goals, implementation route, and timing.	*Chinese Expert Consensus on Perioperative Nutritional Management in Gastrointestinal Surgery (2021 Edition).*
15	Summarize the key pharmaceutical monitoring points for enteral and parenteral nutrition preparations.	*Clinical Pharmacy Consensus on Parenteral Nutrition (2nd Edition)* and *Clinical Pharmacy Consensus on Enteral Nutrition (2nd Edition).*
16	What are the target glucose levels and management principles for postoperative hyperglycemia during the perioperative period of gastric cancer?	Meng Y et al. *Interpretation of the 2020 Expert Consensus on Perioperative Glycemic Management.*
17	How should insulin or oral hypoglycemic therapy be adjusted for diabetic patients undergoing gastric cancer surgery?	*Guidelines for Perioperative Medication Management in Chronic Disease Patients.*
18	What are the key components of perioperative blood pressure evaluation and management in gastric cancer surgery?	*Expert Consensus on Perioperative Hypertension Management. Guidelines for Perioperative Blood Pressure Assessment and Management in Adult Noncardiac Surgery.*
19	How should the risk of postoperative nausea and vomiting (PONV) be assessed preoperatively in gastric cancer surgery, and what are the prevention strategies for high-risk patients?	*Guidelines for Prevention and Treatment of Chemotherapy-Induced Nausea and Vomiting.*
20	What are the key pharmaceutical monitoring points for inhalation therapy during the perioperative period of gastric cancer?	*Expert Consensus on Rational Use of Nebulized Inhalation Therapy (2024 Edition).*

No.	Question	Reference Source
**Section 3. Individualized Medication Cases**		
21	Male, 58 years old, postoperative anastomotic leakage with thoracoabdominal infection and loculated effusion. Provide a consultation on antimicrobial adjustment and assess the need for surgical debridement.	Clinical pharmacy internship assessment question bank
22	Male, 52 years old, with HIV and gastric adenocarcinoma scheduled for radical gastrectomy. Evaluate perioperative management and intraoperative considerations.	Clinical pharmacy internship assessment question bank
23	Female, 66 years old, multiple surgeries and postoperative abdominal infection due to suspected anastomotic leakage. Evaluate whether escalation to ertapenem is appropriate based on microbiological findings.	Clinical pharmacy internship assessment question bank
24	Patient with multiple metastatic gastric cancer, post-splenectomy thrombocytosis (platelet count 567×10^9/L). Assess whether aspirin 100 mg/day is appropriate for thromboprophylaxis.	Clinical pharmacy internship assessment question bank
