# Supplementary material for: Integrating large language models into clinical pharmacy education: applications in perioperative medication management for gastric cancer
Source: Front Med (Lausanne). 2025 Dec 18;12:1710500. doi: 10.3389/fmed.2025.1710500 (PMC12756437; doi:10.3389/fmed.2025.1710500)
Supplement: Supplementary file 2 [file Table_1.DOCX]

**Supplementary Appendix**

Table S1. Types of drug-related problems（DRPs）

| **Primary Domain** | **Primary Domain** | **Primary Domain** | **Quantity** | **Percentage(%)** |
| --- | --- | --- | --- | --- |
| **1.Treatment effectiveness**  There is a (potential) problem with the (lack of) effect of the pharmacotherapy | **P1.1** | No effect of drug treatment despite correct use | 7 | 10.18% |
|  | **P1.2** | Effect of drug treatment not optimal | 23 | 33.6% |
|  | **P1.3** | Untreated symptoms or indication | 18 | 26.42% |
| **2. Treatment safety**  Patient suffers, or could suffer, from an adverse drug event. *N.B*. If there is no specific cause, skip Causes coding | **P2.1** | Adverse drug event (possibly) occurring | 14 | 20.37% |
| **3. Other** | **P3.1** | Unnecessary drug-treatment | 4 | 6.65% |
|  | **P3.2** | Unclear problem/complaint. Further clarification | 2 | 2.78% |

| **Primary Domain** | **Code V9.1** | **Cause** | **Quantity** | **Percentage(%)** |
| --- | --- | --- | --- | --- |
| **1. Drug selection**  The cause of the (potential) DRP is related t of the drug (by patient or o the selection health professional) | **C1.1** | Inappropriate drug according to guidelines/formulary | 12 | 10.43% |
|  | **C1.2** | No indication for drug | 2 | 1.74% |
|  | **C1.5** | No or incomplete drug treatment in spite of existing indication | 27 | 23.48% |
| **3. Dose selection**  The cause of the DRP is related to the selection of the dose or dosage | **C3.1** | Drug dose too low | 5 | 4.35% |
|  | **C3.3** | Dosage regimen not frequent enough | 53 | 46.09% |
| **4. Treatment duration**  The cause of the DRP is related to the duration of treatment | **C4.1** | Duration of treatment too short | 8 | 6.96% |
|  | **C4.2** | Duration of treatment too long | 2 | 1.74% |
| **8. Patient transfer**  **related**  The cause of the DRP can be related to the transfer of patients between primary, secondary and tertiary care, or transfer within one care institution. | **C8.1** | Medication reconciliation problem | 6 | 5.22% |

Table S2. Reasons for drug-related problems（DRPs）

Table S3. Evaluation Question Set for Perioperative Medication Management in Gastric Cancer (24 Items)

| **No.** | **Question** | **Reference Source** |
| --- | --- | --- |
| **Section 1. Incompatibilities and Drug Interactions** | | |
| 1 | As a clinical pharmacist, describe the incompatibilities of cefuroxime sodium for injection and explain the rationale for each category. | Esseti Farmaceutici S.r.l. Cefuroxime Sodium for Injection, Drug Insert. |
| 2 | As a clinical pharmacist, describe the incompatibilities of ceftazidime for injection, categorized by reaction type and underlying mechanism. | GlaxoSmithKline Manufacturing S.p.A. Ceftazidime for Injection, Drug Insert. |
| 3 | As a clinical pharmacist, describe the incompatibilities of cefotaxime sodium for injection, categorized by reaction type and cause. | Xiangbei Wellman Pharmaceutical Co., Ltd. Cefotaxime Sodium for Injection, Drug Insert. |
| 4 | As a clinical pharmacist, summarize the incompatibilities of ceftriaxone sodium for injection, emphasizing ionic incompatibilities and contraindicated diluents. | Shanghai Roche Pharmaceutical Co., Ltd. Ceftriaxone Sodium for Injection, Drug Insert. |
| 5 | As a clinical pharmacist, identify the incompatibilities of cefoperazone–sulbactam for injection and explain the rationale for each category. | Pfizer Inc. Cefoperazone–Sulbactam for Injection, Drug Insert. |
| 6 | As a clinical pharmacist, describe the drug interactions of vancomycin hydrochloride for injection and specify implicated agents and management strategies. | VIANEX S.A.—Plant C. Vancomycin Hydrochloride for Injection, Drug Insert. |
| 7 | As a clinical pharmacist, describe the drug interactions of meropenem for injection and specify implicated agents and management strategies. | Sumitomo Pharma Co., Ltd., Oita Plant. Meropenem for Injection, Drug Insert. |
| **Section 2. Therapeutic Regimens and Monitoring Essentials** | | |
| 8 | Develop a perioperative prophylactic antibiotic regimen for a patient undergoing elective radical gastrectomy (Class II incision). | Guiding Principles for Clinical Application of Antimicrobial Agents (2015 Edition). |
| 9 | Formulate a perioperative anticoagulant management plan, including preoperative withdrawal, bridging therapy, and postoperative resumption. | Guidelines for the Prevention and Management of Perioperative Thrombosis in General Surgery. |
| 10 | How should perioperative management be approached in gastric cancer patients receiving long-term antithrombotic therapy? | Multidisciplinary Expert Consensus on Perioperative Management of Antithrombotic Therapy. |
| 11 | How should postoperative venous thromboembolism (VTE) prevention and management be performed in gastric cancer surgery? | Same as above. |
| 12 | Develop a comprehensive perioperative pain management strategy for gastric cancer patients, including key pharmacological monitoring points. | Expert Consensus on Postoperative Pain Management in Adults. |
| 13 | How should analgesic dosages be adjusted for gastric cancer patients with hepatic or renal impairment during the perioperative period? | Guidelines for Clinical Pharmacist Postoperative Pain Management. |
| 14 | Establish a comprehensive perioperative nutritional management process for gastric cancer patients, including screening, assessment, nutritional goals, implementation route, and timing. | Chinese Expert Consensus on Perioperative Nutritional Management in Gastrointestinal Surgery (2021 Edition). |
| 15 | Summarize the key pharmaceutical monitoring points for enteral and parenteral nutrition preparations. | Clinical Pharmacy Consensus on Parenteral Nutrition (2nd Edition) and Clinical Pharmacy Consensus on Enteral Nutrition (2nd Edition). |
| 16 | What are the target glucose levels and management principles for postoperative hyperglycemia during the perioperative period of gastric cancer? | Meng Y et al. Interpretation of the 2020 Expert Consensus on Perioperative Glycemic Management. |
| 17 | How should insulin or oral hypoglycemic therapy be adjusted for diabetic patients undergoing gastric cancer surgery? | Guidelines for Perioperative Medication Management in Chronic Disease Patients. |
| 18 | What are the key components of perioperative blood pressure evaluation and management in gastric cancer surgery? | Expert Consensus on Perioperative Hypertension Management. Guidelines for Perioperative Blood Pressure Assessment and Management in Adult Noncardiac Surgery. |
| 19 | How should the risk of postoperative nausea and vomiting (PONV) be assessed preoperatively in gastric cancer surgery, and what are the prevention strategies for high-risk patients? | Guidelines for Prevention and Treatment of Chemotherapy-Induced Nausea and Vomiting. |
| 20 | What are the key pharmaceutical monitoring points for inhalation therapy during the perioperative period of gastric cancer? | Expert Consensus on Rational Use of Nebulized Inhalation Therapy (2024 Edition). |
| **Section 3. Individualized Medication Cases** | | |
| 21 | Male, 58 years old, postoperative anastomotic leakage with thoracoabdominal infection and loculated effusion. Provide a consultation on antimicrobial adjustment and assess the need for surgical debridement. | Clinical pharmacy internship assessment question bank |
| 22 | Male, 52 years old, with HIV and gastric adenocarcinoma scheduled for radical gastrectomy. Evaluate perioperative management and intraoperative considerations. | Clinical pharmacy internship assessment question bank |
| 23 | Female, 66 years old, multiple surgeries and postoperative abdominal infection due to suspected anastomotic leakage. Evaluate whether escalation to ertapenem is appropriate based on microbiological findings. | Clinical pharmacy internship assessment question bank |
| 24 | Patient with multiple metastatic gastric cancer, post-splenectomy thrombocytosis (platelet count 567×10⁹/L). Assess whether aspirin 100 mg/day is appropriate for thromboprophylaxis. | Clinical pharmacy internship assessment question bank |
